# Discordance between the patient’s and physician’s global assessment in rheumatoid arthritis: Data from the REAL study—Brazil

**DOI:** 10.1371/journal.pone.0230317

**Published:** 2020-03-13

**Authors:** Maria Fernanda Brandão Resende Guimarães, Maria Raquel da Costa Pinto, Gustavo Gomes Resende, Carla Jorge Machado, Ana Beatriz Vargas-Santos, Rodrigo Balbino Chaves Amorim, Ana Paula Monteiro Gomides, Cleandro Pires de Albuquerque, Manoel Barros Bértolo, Paulo Louzada Júnior, Isabela Araújo Santos, Rina Dalva Neubarth Giorgi, Nathalia de Carvalho Saciloto, Sebastião Cezar Radominski, Fernanda Maria Borghi, Karina Rossi Bonfiglioli, Henrique Carriço da Silva, Maria de Fátima L. da Cunha Sauma, Marcel Lobato Sauma, Júlia Brito de Medeiros, Ivânio Alves Pereira, Gláucio Ricardo Werner de Castro, Claiton Viegas Brenol, Ricardo Machado Xavier, Licia Maria Henrique Mota, Geraldo da Rocha Castelar-Pinheiro

**Affiliations:** 1 Universidade Federal de Minas Gerais, Belo Horizonte, Brazil; 2 Universidade do Estado do Rio de Janeiro, Rio de Janeiro, Brazil; 3 Universidade de Brasília, Brasília, Brazil; 4 Universidade Estadual de Campinas, Campinas, Brazil; 5 Universidade de São Paulo, Ribeirão Preto, Brazil; 6 Hospital do Servidor Publico Estadual de São Paulo, São Paulo, Brazil; 7 Universidade Federal do Paraná, Curitiba, Brazil; 8 Universidade Estadual de Maringá, Maringá, Brazil; 9 Universidade de São Paulo, São Paulo, Brazil; 10 Universidade Federal do Para, Belém, Brazil; 11 Universidade Federal de Santa Catarina, Florianópolis, Brazil; 12 Universidade do Sul de Santa Catarina-Unisul, Florianópolis, Brazil; 13 Universidade Federal do Rio Grande do Sul, Porto Alegre, Brazil; Universita Campus Bio-Medico di Roma, ITALY

## Abstract

**Background:**

Discordance between patient’s global assessment (PtGA) and physician’s global assessment (PhGA) has been described in rheumatoid arthritis (RA). Understanding the reasons for this discrepancy is important in the context of treat-to-target treatment strategy.

**Objective:**

To assess the determinants of PtGA and PhGA and factors associated with discordance between them.

**Methods:**

The REAL study included RA patients from Brazilian public health centers. Clinical, laboratory and outcomes measures were collected. PtGA and the PhGA were rated on a visual analog scale and analyzed. Three groups were defined: no discordance (difference between PtGA and PhGA within 3 cm), positive discordance (PtGA exceeding PhGA by >3 cm), and negative discordance (PtGA less than PhGA by >3 cm). Multivariate regression analysis was used to identify determinants of PtGA and PhGA and their discordance.

**Results:**

1115 patients (89,4% female, mean age 56.7y and median disease duration of 12.7y) were enrolled. Two factors were associated with PtGA in the final multivariate model: one point increase in the pain scale leads to an increase of 0.62 in PtGA; one point increase in HAQ increases by 9,25 points the PtGA. The factors associated with PhGA were pain scale, number of tender and swollen joints (NTJ and NSJ), positive RF, ESR, HAQ-DI and use of corticosteroids. Discordance between patient and physician was found in 30.52%: positive discordance in 24.6% and negative discordance in 5.92%. An increase of one point in the NSJ was associated with a 12% increase in the chance of negative discordance. The chance of positive discordance increased by 90% and 2% for each unit increased in HAQ-DI and pain scale respectively. Finally, the chance of positive discordance decreased by 3% for each point increased in NTJ and by 15% for each point increased in NSJ.

**Conclusion:**

In one-third of the assessments, there was disagreement between PtGA and PhGA (a positive discordance was found in 80% of them). Pain and function were determinants for patients to estimate disease activity, while swollen joints was the main factor related to a worse physician’s evaluation. These data show how different can be the perspectives of patients and assistants.

## Introduction

Rheumatoid arthritis (RA) is a systemic inflammatory disease characterized by pain and inflammatory involvement of the synovial joints with the potential to lead to joint destruction, functional disability, and reduced quality of life [1].

In RA, the clinical history and physical examination of the patient are important factors that determine clinical decisions [2]. Disease activity indices include global assessments performed by both patients and physicians using visual analog or numerical scales ranging from 0 to 10 cm and are frequently used parameters of RA activity. Disease activity indices include global assessments performed by both patients and physicians using visual analog or numerical scales ranging from 0 to 10 cm and are frequently used parameters of RA activity. The patient’s global assessment (PtGA) usually reflects general evaluation of health status and, while it may be a more subjective measure, it was demonstrated to be a reliable parameter of disease activity. On the other hand, physician’s global assessment (PhGA) tends to reflect the analysis of aspects such as physical exam findings and complementary exams results [3–5]. Both PtGA and PhGA can be considered measures of the same variable: the current inflammatory activity of RA. Therefore, it could be thought that their values would always be similar. However, in several diseases, including RA, significant disagreement between the two measures has been observed, at varying frequencies [3, 6, 7]. In daily care practice, it is often observed that the PtGA is higher than expected based on the clinical activity of the disease [8]. Among patients with RA, discordance with their physicians in the global assessment of disease activity, in which the patient’s assessment is substantially higher, is present in approximately 30% of visits [7, 9–11].

Understanding the reasons for the discrepancies between the PtGA and PhGA has become particularly important in the current context that defines remission as the treatment target [12]. Moreover, there is evidence suggesting that discordance is predictive of low health-related quality of life, loss of work productivity, and impaired functional capacity [6–13].

Considering these issues, the objective of the present study was to evaluate the determinants of the PtGA and PhGA scores and to determine the factors associated with their discrepancies in an observational study of Brazilian patients with RA.

## Materials and methods

The Rheumatoid Arthritis in Real Life (REAL) study is a prospective multicenter observational cohort study with 12 months of follow-up. The objectives of the study were to describe the demographic, clinical, and therapeutic characteristics of Brazilian patients with RA and evaluate their treatment adherence, safety of pharmacological treatment and impact on the quality of life, physical function, and work ability.

Eleven tertiary care public health centers specialized in caring for RA patients were selected to represent the five geographic regions in Brazil. The recruitment period began on August 12, 2015 and ended on April 15, 2016. Patients were followed-up for approximately 12 months with systematic data collection at the initial visit (baseline), intermediate visit (6 months ± 1 month), and final visit (12 months ± 1 month) with an additional descriptive report of any other unscheduled visit. The present study is a cross-sectional evaluation of the data collected during the initial visit [14].

### Participants

The inclusion criteria were as follows: 1) fulfillment of the 1987 American Rheumatism Association (ARA) or the 2010 American College of Rheumatology (ACR) / European League Against Rheumatism (EULAR) classification criteria of the for RA [15, 16]; 2) 18 years of age or older; and 3) documented medical record data from at least six months of follow-up in their health care center before inclusion in the study. Patients were excluded if they could not reliably complete the self-assessment sections of the data collection instrument. Each center included approximately 100 patients consecutively.

Demographic and socioeconomic data, family history of RA, other autoimmune diseases or associated conditions, and personal history of comorbidities and lifestyle (smoking, alcohol consumption, and physical activity) were collected at the initial visit. The following were also evaluated: disease duration, time from symptom onset to diagnosis, time to the first disease-modifying antirheumatic drug (DMARD), health care unit and physician’s specialty at first health care system visit due to RA symptoms, presence of extra-articular manifestations, positivity for rheumatoid factor (RF) and anti-citrullinated protein antibody (ACPA), and presence of bone erosions on X-ray in both hands and both feet.

Additionally, previous pharmacological treatments for RA (with the respective reasons for discontinuation), history of orthopedic surgery, and history of intraarticular or periarticular steroid injections were described.

The clinical assessment included vital signs, anthropometric measurements, number of tender (NTJ) and swollen (NSJ) joints, and medical score on the PhGA. The results reported by the patients included pain, overall health, disease activity assessed by the patient’s global assessment (PtGA), fatigue, morning stiffness, and articular index, in which the patient evaluates the presence of pain and its intensity in 16 joints.

Patients evaluated their level of disease activity on the PtGA from 0 to 10 cm, where 10 was considered the worst possible disease activity (very poor) and 0 was the best disease activity (very well). Patients were asked "considering all the ways that your joint disease affects you, mark an "X" through the line for how well you are feeling." The same physician, on a separate visual analogue scale, performed the PhGA, after finishing the patient’s clinical examination.

A discordance score was calculated by subtracting the PhGA score from the PtGA score. Then, the patients were separated into one of three categories: (1) no discordance when the patient’s and the physician’s assessments were up to 3.0 cm away from each other; (2) negative discordance when the patient’s assessment was underestimated relative to the physician’s (Δ > -3); and (3) positive discordance when the patient’s assessment was overestimated relative to the physician’s assessment (Δ > 3).

The Disease Activity Index-28 Joints based on the erythrocyte sedimentation rate (DAS28(ESR)), Clinical Disease Activity Index (CDAI), Simplified Disease Activity Index (SDAI), and Rheumatoid Arthritis Disease Activity Index (RADAI) were calculated [17–19].

The translated and validated versions of the Health Assessment Questionnaire-Disability Index (HAQ-DI), Short Form-12 (SF-12), and Short Form-6 Dimensions (SF-6D) were used to evaluate physical function, functional capacity and well-being, and health status from the patient’s perspective, respectively [20–22].

Laboratory results of ESR and C-reactive protein (CRP) were recorded.

### Statistical analysis

The mean and standard deviations were calculated as well as the median and interquartile range (IQR: 25th percentile-75th percentile) for continuous variables. The median and IQR were calculated only for variables with a non-normal distribution based on the Shapiro-Wilk test (p<0.05). Frequencies and proportions were obtained for categorical variables.

To assess the association between the PtGA and the variables selected for the study and between the PhGA and these same variables, we used quantile regression, which is more robust in the presence of outliers than traditional linear regression by least squares. In the quantile regression, coefficients less than zero indicate a negative association; if they are above zero, the association is positive. Univariate regression was used, and variables found to be significant by the Wald test at the 20% level (p<0.20) were selected to construct a multivariate model. Next, the variables with significance levels above 5% were removed from the model, leaving only those with significance levels below 5% (p<0.05) in the final model. Confidence intervals were also calculated.

To assess the association between positive and negative discordances, a multinomial logistic model was used in which the reference category for the response variable was the absence of discordance (between -3 and +3). Odds ratios were calculated for the univariate and multivariate analyses. The final model construction process followed that indicated for analysis of the scales. The 95% confidence intervals were also calculated.

### Ethics statement

This study was approved by the National Research Ethics Commission (Comissão Nacional de Ética em Pesquisa—CONEP)—Ministry of Health and by the local Research Ethics Committee (Comissão de Ética em Pesquisa–COEP)–Federal University of Minas Gerais. All patients signed an informed consent form.

## Results

A total of 1,115 patients were included in the study. The demographic and general clinical data of the population at the time of the initial assessment are shown in Tables 1 and 2. Approximately 90% were female, with a mean age of 56.7 years and a median disease duration of 12.7 years. The median years of education was 8, and 3.23% of subjects were below the literacy level.

**Table 1:** Baseline demographic data of patients enrolled in the REAL study.

Demographic data	Absolute value or %	N
Age, years, median (range)	56.7 (22.1–88.8)	1115
Female gender, %	89.4	1115
Ethnicity/race/color, %		1115
White	56.8	
Pardo*	31.3	
Black	10.9	
Others	1.0	
Smoking, %		1115
Smoker	10.9	
Former smoker	28.6	
Never smoked	60.5	
BMI categories, %		1046
Low weight	5.0	
Normal	31.5	
Overweight	35.3	
Obesity	28.2	
Total formal education time, years, median (range)	8 (0–20)	1075
Brazilian Economic Classification Criterion: Socioeconomic Strata: Gross family income in the month in US dollar**, %		1101
A (5,921.00)	1.4	
B1 (2,623.00)	3.5	
B2 (1,357.00)	18.4	
C1 (766.81)	27.4	
C2 (460.65)	31.3	
D-E (217.71)	18.0	

*Mixed white and black ethnicities. BMI: Body mass index.

** Conversion of Brazilian Reais into US dollars made in accordance with the exchange rate of April 16, 2016—US$1,00: R$ 3,5276

**Table 2:** Baseline clinical data of patients enrolled in the REAL study.

Clinical Data	Absolute value or %	n
Disease duration, years, median (range)	12.7 (0.7–56.9)	1114
Early disease (≤24 months)	3.59%	40
Intermediary duration disease (>24mo and ≤60mo)	10.95%	122
Late disease (>60 months)	89.05%	992
Time from symptoms to diagnosis, months, median (range)	12 (1–457)	1078
Time from symptoms to 1^st^ DMARD, months, median (range)	12 (1–624)	994
Patients with ≥1 extra-articular manifestation, %	23.3	1115
Positive rheumatoid factor, %	78.2	1105
Positive anti-citrullinated peptide antibody, %	77.2	477
Erosive disease, %	54.9	1095
Fibromyalgia, %	13.8	1115
Patients fulfilling classification criteria, %:		
ARA 1987	90.0	1115
ACR/EULAR 2010	90.9	1115
Both	80.8	1115
Drugs in use, %:		
Glucocorticoids	47.4	1115
Nonsteroidal anti-inflammatory drugs	9.1	1115
Synthetic DMARD	90.9	1115
Methotrexate	66.5	1115
Biologic DMARD	35.7	1115
Biologic DMARD in monotherapy	5,6	1115
ESR, median (range)	21 (1–140)	923
C-reactive protein, median (range)	0.7 (0–76.1)	944
Pain (VAS 0–100), median (range)	40 (0–100)	1115
Fatigue (VAS 0–100), median (range)	40 (0–100)	1115
Global health assessment (VAS 0–100), median (range)	38 (0–100)	1115
DAS28(ESR), median (range)	3.5 (0.3–8.2)	923
Remission	26.2	
Low disease activity	15.1	
Moderate disease activity	41.8	
High disease activity	16.9	
CDAI, median (range)	9 (0–70)	1113
Remission	20.1	
Low disease activity	33.2	
Moderate disease activity	27.5	
High disease activity	19.2	
HAQ-DI, median (range)	0.875 (0–3)	1111
SF-12 physical, median (range)	36.1 (17.5–55.9)	1079
SF-12 mental, median (range)	47.1 (14.3–72.0)	1079

ARA: American Rheumatism Association; ACR: American College of Rheumatology; EULAR: European League Against Rheumatism; DMARD: disease-modifying antirheumatic drug; ESR: erythrocyte sedimentation rate; VAS: visual analog scale; DAS28: Disease Activity Score 28-joint count; CDAI: Clinical Disease Activity Index; HAQ-DI: Health Assessment Questionnaire-Disability Index; SF-12: 12-Item Short-Form Health Survey.

The interval between symptoms onset and diagnosis ranged from 1 to 457 months (median of 12 months).

The median HAQ-DI score was 0.875, ranging from 0 to 3. The median DAS28-ESR score was 3.5, and 58.7% of patients presented with moderate or high disease activity. When evaluated by the CDAI, 46.7% of the individuals were classified as presenting with moderate to high disease activity (median = 9).

Almost half of the patients used glucocorticoids; among them, 96.5% used DMARDs, and 35.7% used biologics [14].

Regarding the PtGA, only two factors were associated in the final multivariate model. While a one-point increase on the pain scale leads to a 0.62-point increase in the PtGA score, a one-point increase on the HAQ-DI increases the PtGA score by 9.25 points. In the PhGA, several factors showed significant influence after the multivariate analysis, namely, pain scale, NSJ and NTJ, positive RF, ESR, HAQ-DI score, and use of corticosteroids (Tables 3 and 4).

**Table 3:** Factors influencing the PtGA and PhGA—Univariate analysis.

Variables	Patient Assessment	Physician Assessment
	Coefficient (95% CI)	Coefficient (95% CI)
Pain Scale	0.78 (0.72; 0.84)***	0.49 (0.45; 0.53)***
NSJ (0 to 28)	2.38 (1.71; 3.05)***	4.83 (4.22; 5.44)***
NTJ (0 to 28)	1.67 (1.32; 2.02)***	2.38 (2.11; 2.65)***
Male gender (ref: female)	-4 (-15.4; 7.44)	-1 (-5.69; 3.70)
Years of study	-1.07 (1.97; -0.17)*	-0.11 (-0.36; 0.14)
Months of symptoms	0.04 (0.01; 0.06)*	0.01 (0.00; 0.02)*
Rheumatoid factor (ref: negative)		
--Low-positive	4 (-6.81; 14.8)	1 (-3.51; 5.51)
--High-positive	9 (0.07; 18.1) =	4 (0.25; 7.75)*
ESR	0.04 (-0.16; 0.25)	0.18 (0.99; 1.00)***
CRP	1.15 (0.02; 2.29)*	1.26 (0.97; 1.54)***
HAQ-DI	23.3 (20.2; 26.5)***	15.2 (13.6; 16.8)***
Fibromyalgia	-4 (-12.7; 4.71)	-1 (-5.17; 3.17)
Age	0.26 (-0.11; 0.64) 0.2	0.03 (-0.10: 0.16)
Corticosteroids	19 (16.0; 22.0)***	10 (8.08; 11.9)***
Anti-inflammatories	14 (-1.20; 29.2) =	10 (6.76; 13.2)***

*** p<0.001;

** p<0.01;

* p<0.05; = p<0.1; 0.2 p<0.2.

HAQ-DI: Health Assessment Questionnaire-Disability Index;

**** Negative refers to IU values that are less than or equal to the upper limit of normal (ULN) for the laboratory and assay; low-positive refers to IU values that are higher than the ULN but 3 times the ULN for the laboratory and assay; high-positive refers to IU values that are 3 times the ULN for the laboratory and assay.

**Table 4:** Factors influencing the PtGA and PhGA—Multivariate analysis.

Variables	Patient Assessment	Physician Assessment
	Coefficient (95% CI)	Coefficient (95% CI)
Pain scale	0.62 (0.55; 0.68)***	0.23 (0.19; 0.27)***
NSJ (0 to 28)	---	2.37 (2.07; 2.67)***
NTJ (0 to 28)	---	0.95 (0.80; 1.11)***
Rheumatoid factor (ref: negative)	---	
--Low-positive	---	3.96 (1.26; 6.67)**
--High-positive	---	2.42 (0.16; 4.68)*
ESR	---	0.08 (0.04; 0.12)***
HAQ-DI	9.25 (6.69; 11.1)***	2.98 (1.54; 4.41)***
Use of corticosteroids	---	2.36 (0.52; 4.21)*

*** p<0.001;

** p<0.01;

* p<0.05; = p<0.1

HAQ-DI: Health Assessment Questionnaire-Disability Index;

Discordance between the global scales with variation greater than 3 points occurred in 30.52% of the patients evaluated. Of these, 5.92% had negative discordance scores, i.e., the PtGA score was lower than the PhGA score, and 24.6% had positive discordance scores with higher PtGA scores.

NSJ was significantly associated with the negative discordance score after the final multivariate analysis. A one-point increase in the NSJ was associated with a 12% increase in the odds of negative discordance (compared to the absence of discordance). In the case of positive discordance, four factors remained associated: the pain scale and the HAQ-DI in a positive manner, and the NSJ and NTJ in a negative manner. A one-point increase in the HAQ-DI score was associated with a 90% increase in the odds of positive discordance (compared to the absence of discordance), whereas a one-point increase on the pain scale was associated with a 2% increase in the odds of positive discordance. In the case of the NTJ, a one-point increase was associated with a decrease of 3% in the odds of positive discordance (OR = 0.97), and this decrease was 15% in the case of the NSJ (OR = 0.85) (Tables 5 and 6).

**Table 5:** Factors influencing the discordance score—Univariate analysis.

Variables	Negative discordance (PtGA-PhGA) < (-3.0) Physician’s higher than patient’s		Positive discordance (PtGA-PhGA) > (+3.0) Physician’s lower than patient’s	
	OR (95% CI)	P-value	OR (95% CI)	P-value
Pain Scale	1.01 (1.00; 1.02)	0.020	1.02 (1.01; 1.02)	<0.001
NSJ (0 to 28)	1.14 (1.09; 1.20)	<0.001	0.93 (0.89; 0.98)	0.003
NTJ (0 to 28)	1.06 (1.03; 1.09)	<0.001	0.99 (0.97; 1.02)	0.540
Male gender (ref: female)	1.22 (0.58; 2.54)	0.604	0.61 (0.37; 1.01)	0.052
Years of study	0.94 (0.89; 1.00)	0.071	0.96 (0.93; 1.00)	0.028
Months of symptoms	1.00 (1.00; 1.00)	0.729	1.00 (1.00; 1.00)	0.015
Rheumatoid factor (ref: negative)				
--Low-positive	1.09 (0.51; 2.34)	0.822	0.73 (0.48; 1.12)	0.153
--High-positive	1.01 (0.52; 1.95)	0.979	0.85 (0.60; 1.20)	0.349
ESR	1.02 (1.01; 1.03)	<0.001	1.00 (0.99; 1.00)	0.341
CRP	1.03 (0.98; 1.08)	0.205	1.01 (0.98; 1.05)	0.472
HAQ-DI	1.78 (1.29; 2.44)	<0.001	1.85 (1.55; 2.22)	<0.001
Fibromyalgia	0.75 (0.34; 1.70)	0.501	1.11 (0.75; 1.64)	0.604
Age	1.00 (0.98; 1.02)	0.919	1.02 (1.00; 1.03)	0.016
Corticosteroids	1.36 (0.82; 2.25)	0.231	1.29 (0.98; 1.71)	0.065
Anti-inflammatories	1.09 (0.45; 2.62)	0.846	1.34 (0.85; 2.12)	0.208

NSJ = number of swollen joints; NTJ = number of tender joints; ESR = erythrocyte sedimentation rate; CRP = C-reactive protein; HAQ-DI: Health Assessment Questionnaire-Disability Index

**Table 6:** Factors influencing the discordance score—Multivariate analysis.

Variables	Negative discordance (PtGA-PhGA) < (-3.0) Physician’s higher than patient’s		Positive discordance (PtGA-PhGA) > (+3.0) Physician’s lower than patient’s	
	OR (95% CI)	P-value	OR (95% CI)	P-value
Pain Scale	1.00 (0.99; 1.01)	0.854	1.02 (1.01; 1.02)	<0.001
NSJ (0 to 28)	1.12 (1.04; 1.18)	<0.001	0.85 (0.80; 0.91)	<0.001
NTJ (0 to 28)	1.03 (1.00; 1.06)	0.155	0.97 (0.94; 0.9995)	0.046
HAQ-DI	1.18 (0.79; 1.76)	0.428	1.90 (1.51; 2.38)	<0.001

NSJ = number of swollen joints; NTJ = number of tender joints; ESR = erythrocyte sedimentation rate; HAQ-DI: Health Assessment Questionnaire-Disability Index

## Discussion and conclusions

In this study, we found discordance equal to or greater than 3 points between the disease activity scales reported on the same day by patients and their physicians in 30.52% of the cases. The cut-off point of 3 was chosen based on published studies, including a recent meta-analysis that showed that this is the most used discordance cutoff in the literature [7]. Occurrences of the patient’s disease activity assessment surpassing that of the physician were much more frequent than the opposite (24.6% vs. 5.92%). These data are in strong agreement with the majority of studies published on the same topic in RA (even considering different cutoffs for the difference between assessments), in which positive discordances (patient’s assessment worse than physician’s) were found in 18 to 49% of cases, while negative discordances (physician’s assessment worse than patient’s) were much less frequent in 3 to 9% of cases [3, 6, 9–11, 13, 23–25].

Among the possible determinants of these discrepancies, we found that pain (measured on a 0–10 analog scale) and physical function (measured on a 0–3 scale in the HAQ-DI) contributed more to the PtGA; joint count (especially the NSJ), positive rheumatoid factor, physical function, and use of corticosteroids influenced the PhGA most. Physical function, which was the only variable associated with the two scales in the multivariate model, exerted greater influence on the patient’s scale (coefficient 9.25/point on the HAQ-DI) than on the physician’s scale (coefficient 2.98/point on the HAQ-DI). Together, these data suggest a difference in perspectives among those involved. Physicians tend to give greater weight to objective variables, such as those observed during the physical examination, while patients give greater significance to the subjective aspects of the assessment, especially pain. This distance between points of view was also found in several other studies. In general, pain and/or physical function were associated with poorer patient assessment in the vast majority of studies [3, 8, 10, 11, 13, 23–29], whereas NSJ and/or abnormal acute inflammation tests were more frequent determinants of worse physician’s assessments [8–11,13, 26, 27, 29].

Numerous potential causes for this discordance have been identified, particularly pain due to inflammatory and non-inflammatory processes different from RA, fatigue, functional disability, depression, psychological stress, low health literacy, and patient-physician communication problems [6, 9, 10, 26]. In the present study, fibromyalgia was not associated with discordance (negative or positive) between PtGA and PhGA, contrary to the findings of other studies [9, 13, 23]. This may be due to how this variable was obtained–by reviewing the medical records–, which may not be accurate to reflect the state of this comorbidity by the time of the interview. Nevertheless, this lack of association has already been shown in patients with RA [7] and also in early spondyloarthritis [24].

A limitation of this study was that it has included patients with different stages of disease activity and a great proportion of patients with late disease, which could interfere with some results. As it can be seen in Table 2, the sample composition regarding disease duration has an imbalance, with a great predominance of patients with more than five years of disease duration– 89% of the study population–, which did not allow intergroup comparisons.

Longitudinal studies have shown a negative effect on health-related quality of life [13] and a greater impact on work productivity [6] among patients who persisted with worse disease activity scores than those of their physicians.

A better understanding of factors associated with discordance between physicians and patients in the management of RA can lead to a better doctor-patient relationship, which facilitates shared decisions. Share decisions are currently highly promoted and can improve patient satisfaction with their disease management and treatment adherence, possibly leading to better long-term outcomes. Future studies with appropriate designs should be performed to clarify the extent to which these unfavorable outcomes can be intervened with by bringing together the perspectives and expectations of patients and physicians involved in RA management.
